# A Phase I Trial to Determine the Safety and Tolerability of Autophagy Inhibition Using Chloroquine or Hydroxychloroquine in Combination With Carboplatin and Gemcitabine in Patients With Advanced Solid Tumors

**DOI:** 10.3389/fonc.2022.811411

**Published:** 2022-04-26

**Authors:** Nagla Abdel Karim, Asad Ullah, Imran Ahmad, Elmustapha Bahassi, Olugbenga Olowokure, Ahmed Khaled, Harold Davis, John C. Morris

**Affiliations:** ^1^ Division of Hematology/Oncology-Augusta University, Augusta, GA, United States; ^2^ Division of Hematology/Oncology-The University of Cincinnati, Augusta, GA, United States; ^3^ GlaxoSmithKline, Division of Solid Tumors-Augusta, Augusta, GA, United States

**Keywords:** lung cancer, autophagy, phase 1, solid tumor, chloroquine

## Abstract

**Background:**

Autophagy is a catabolic process that is triggered in cells during periods of metabolic or hypoxic stress, which enables their survival during this challenge. Autophagy may also impart survival advantage to tumors cells undergoing attack from chemotherapy or radiation. Inhibition of early-stage autophagy can rescue cancer cells, while inhibition of late-stage autophagy enhances cell death due to accumulation of damaged organelles. The antiparasitic drugs chloroquine (CQ) and hydroxychloroquine (HCQ) inhibit late-phase autophagy. We assessed the safety, tolerability, and efficacy of combining CQ or HCQ with carboplatin and gemcitabine (CG) in patients with refractory advanced solid tumors.

**Methods:**

This single institution phase 1 dose-escalation study was designed to evaluate the maximum tolerated dose (MTD) of CQ/HCQ, in combination with CG, in patients with advanced solid tumors. Secondary objectives were to determine overall response rate (ORR), progression-free survival (PFS), and overall survival (OS). A starting dose of CQ or HCQ 50 mg was used in conjunction with standard starting doses of CG and increased in increments of 50 mg in each patient dose cohort. Grade 3 or greater toxicity that is treatment related, and was not self-limited, or not controlled in <7 days was considered dose-limiting toxicity (DLT).

**Results:**

Twenty-two patients were enrolled. All patients had at least one prior treatment, and 11 of them had 3 prior regimens. CQ/HCQ 100 mg daily was found to be the MTD in combination with CG with thrombocytopenia and/or neutropenia dose limiting. The median overall (OS) was 11 months, and the 1- and 3-year OS were 30% and 7%, respectively. Median progression-free survival was 5 months, and the 6-, 12-, and 18-month progression-free survivals were 48%, 21%, and 14%, respectively.

**Conclusion:**

The MTD identified for CQ/HCQ was lower than previously reported with concomitant use of chemotherapeutic regimes likely due to the myelosuppressive nature of CG in previously treated patients.

## Introduction

Autophagy, or “self-eating,” is a cellular process by which cytoplasmic organelles and proteins are sequestered into autophagic vesicles and delivered to lysosomes for “bulk” degradation and recycling (1, 2). It is a housekeeping process that regulates organelle and cellular protein turnover (3). Autophagy has been shown to become deregulated in certain pathological states including cancer. Under normal circumstances, autophagy is believed to suppress cellular transformation and tumor progression by limiting chromosomal instability. Alternatively, it has been demonstrated that established tumors utilize autophagy to survive periods of metabolic or hypoxic stress (4). Thus, manipulation of autophagy has become a potential area for the development of novel antineoplastic strategies (5). Aminoquinolines such as CQ have been shown to inhibit autophagy by mechanisms distinct from other inhibitors such as 3-methyladenine (3-MA). Whereas 3-MA inhibits early phase autophagy, consequently inhibiting the formation of acidic vesicular organelles (AVOs) that consist predominantly of autophagosomes and autolysosomes, CQ inhibits autophagy in its late phases after cytoplasmic AVOs have been formed. Therefore, CQ-treated cells typically demonstrate accumulation of cytoplasmic AVOs (6). CQ has been identified as a chemotherapy sensitizer when used in combination with certain antineoplastic drugs (7, 8). The lysosomotropic properties of CQ are likely responsible for many of its biological effects. Accumulating lines of evidence suggest that through its lysosomotropic effect, CQ can sensitize cancer cells to the killing effects of and various chemotherapeutic agents and ionizing radiation (9, 10).

In a small, randomized study, Sotelo et al. (5), reported improved survival in patients with glioblastoma treated with four cycles of carmustine with radiation and CQ versus placebo beginning 5 days after surgery.

Amaravadi et al. demonstrated that targeting autophagy with CQ derivatives enhanced the efficacy of chemotherapy (7) HCQ has been extensively studied in combination with several chemotherapeutic agents to assess its clinical safety and activity. Several phase I trials studying HCQ in combination with various antineoplastic agents determined the maximum tolerated dose (MTD) to be 200–1,200 mg daily. HCQ has been studied in combination with temozolomide 150 mg in patients with advanced solid tumors (11). Wolpin et al. reported the safety and antineoplastic activity of HCQ in 20 patients with metastatic pancreatic cancer who did not respond to conventional chemotherapy. In this phase II trial, patients received 400 mg (*n* = 10) or 600 mg (*n* = 10) of HCQ twice daily as a single agent (12).

Five other phase I trials of HCQ involved combination with various chemotherapeutic agents including temozolomide, bortezomib, temsirolimus, vorinostat, or doxorubicin (7, 12, 13). A number of patients with melanoma, colorectal cancer, myeloma, and renal cell carcinoma demonstrated partial responses or stable disease, suggesting antitumor activity. In a phase II study in advanced pancreatic cancer, Karasic et al. showed that HCQ 600 mg daily in combination with gemcitabine and nab-paclitaxel resulted in an improved response rate, making some tumors resectable (14). Based on this rationale and the importance of gemcitabine and carboplatin in treating many types of cancer, our study was designed to investigate if CQ will resensitize use of chemotherapy again in heavily pretreated patients. Patients enrolled in our phase I study were mostly heavily pretreated and were candidates for the systemic therapy with carboplatin and gemcitabine and thus the choice of starting with lower doses of HCQ.

## Study Objectives

### Primary Objective

This study primarily aims to determine the maximum tolerated dose (MTD) of chloroquine (CQ) or (HCQ) in combination with carboplatin and gemcitabine (CG) in patients with advanced solid tumors.

### Secondary Objectives

The secondary objectives were as follows:

1. To estimate the overall response rate (ORR), progression-free survival (PFS), and overall survival (OS) of patients with advanced solid tumors treated with chloroquine (CQ) or (HCQ). HCQ has been used in place of CQ due to the acute shortage in the US and since both has similar chemistry and efficacy.
2. To determine the pharmacokinetics of CQ/HCQ in combination with CG; and
3. To detect effects on autophagy through changes in plasma levels of exosomal microtubule-associated protein 1A/1B light chain 3B (LC3) levels in peripheral blood.

## Patients and Methods

### Human Subjects Protections

Eligible patients were enrolled in this Institutional Review Board (IRB)-approved study through the University of Cincinnati Cancer Institute Clinical Trials Office (CTO). To register a patient, all of the following were obtained: written informed consent form, Health Insurance Portability and Accountability Act (HIPAA) Authorization form, eligibility screening worksheet, and registration form. The trial was listed in https://clinicaltrials.gov (NCT02071537).

### Study Design

This was a single institution phase I dose-escalation study using a 3 + 3 dose-escalation schema. Patients with progressing advanced solid tumors with either no other available standard of care treatment or where carboplatin and gemcitabine were considered an acceptable treatment option, Eastern Cooperative Oncology Group (ECOG) performance status 0–1 were eligible. Sequential CQ/HCQ dose cohorts of three to six patients were treated. The starting dose of CQ was 50 mg p.o. days 1–21 in addition to intravenous carboplatin (AUC 5) and gemcitabine (500 mg/2) day 1 (**Table 1**). Patients in cohort 1 were treated with CQ; however, CQ became unavailable due to an international shortage, so the study continued using HCQ at the cited doses in cohorts 2 and 3 and the expansion cohort with IRB-approval.

**Table 1:** Planned dose escalation and MTD cohort expansion.

Dose level	Patients	CQ (first cohort) HCQ (all subsequent patients) (mg/day)	Carboplatin (AUC)	Gemcitabine (mg/m^2^)
		Day −7 to day 21	Day 1	Day 1 and 8 out of 21-day cycle
1	3–6	CQ 50 mg daily	5	1,250-1,000
2	3–6	HCQ 100 mg daily	5	1,000
3	3–6	HCQ	5	1,000
4	3–6	200	5	1,000
Expansion cohort	10–12	100	5	1,000

### Eligibility Criteria

Subjects were required to have histologically or cytologically confirmed metastatic or unresectable cancer for which either standard curative measures do not exist, are no longer effective, or for which the combination of carboplatin and gemcitabine are considered a reasonable treatment option; no other than active malignancy, or chronic systemic immune therapy, and no known G-6-PD deficiency; age ≥18 years; ECOG performance status <2 (Karnofsky >60%); acceptable organ and bone marrow function defined as an absolute neutrophil count ≥1,500/µl, platelet count ≥100,000/µl, total bilirubin <1.5× upper limit of normal (ULN), aspartate aminotransferase (AST) [serum glutamic-oxaloacetic transaminase (SGOT)], or alanine aminotransferase (ALT) [serum glutamic-pyruvic transaminase (SGPT)] <3× ULN; adequate baseline renal function with serum creatinine <1.5× ULN; a life expectancy >3 months; and at least one measurable lesion by RECIST 1.1. Patients with treated and asymptomatic brain metastases were eligible. Women and men of child-bearing potential must have agreed to use adequate contraception for the duration of study, and participants must have the ability to understand and willingness to sign a written informed consent document. Patients receiving other investigational agents, those with untreated brain metastases, history of allergic reactions to CQ/HCQ or other agents used in study, and an uncontrolled intercurrent illness or infection were ineligible.

### Treatment

CQ or HCQ was administered at the dose levels, as indicated in **Table 1**, for a total of four 21-day treatment cycles (initially HCQ was used, then due to unavailability of HCQ, patients were switched to CQ). CQ was administered orally daily starting 1 week prior to the start of carboplatin and gemcitabine (CG) chemotherapy (day −7 until day 1) andthroughout the 21-day cycle for a total of four treatment cycles of CG. Additional fifth and sixth cycles of carboplatin and gemcitabine were allowed without the addition of CQ or HCQ in case of continued response or benefit per the decision of the treating investigator. The lower and higher dose groups (N = 6 and 3, respectively) received 50 or 150 mg of CQ or HCQ as a fixed daily oral dose. The first seven patients received CQ 50 mg; 50 mg was given in a suspension form, then 100 mg was given through splitting the 200-mg tablet (where the first patient received only one dose of 50 mg of CQ and was found to be ineligible after dosing on day 1 and was excluded and replaced), and the next three patients received 100 mg of HCQ due to the worldwide shortage and unavailability of CQ. The third cohort received 150 mg of HCQ and the expansion cohort of 10 patients received 100 mg of HCQ. HCQ tablets were split into half to provide the 100-mg dose. This was done by an experienced clinical pharmacist to ensure all patients are getting the same dose. For the dose-limiting toxicity definition and dose escalation, the dose-limiting toxicity (DLT) of HCQ was 150 mg when given in combination with carboplatin and gemcitabine. We believe that the major toxicity though occurred due to the cytotoxic chemotherapy in heavily pre-treated patients. The maximum tolerated dose (MTD) of HCQ was 100 mg when given in combination with carboplatin and gemcitabine.

### Evaluation of Safety and Outcome

Adverse event descriptions and grading as described in the revised National Cancer Institute (NCI) Common Terminology Criteria for Adverse Events (CTCAE) version 4.0 were utilized for AE reporting (CTCAE 4.0 was the available criteria used during the evaluation of our studied patients). Primary outcome measures were as follows: CTCAE grade >3 adverse events clearly linked to treatment and was not self-limited or resolved in <7 days. Secondary outcome measures were RECIST 1.1 response criteria: complete response (CR), partial response (PR), stable disease (SD), and progressive disease (PD). The duration of overall response was measured from the time that the measurement criteria are met for CR or PR (whichever is first recorded) until the first date that recurrent or progressive disease was objectively documented. Duration of stable disease is measured from the start of the treatment until criteria for progression are met, taking as reference the smallest RECIST measurements recorded since the treatment began. Progression-free survival (PFS) is defined as the duration of time from start of treatment to time of progression.

### Ocular Exam

Due to the potential ocular toxicity of CQ/HCQ, all subjects underwent a baseline ocular/funduscopic exam before the start of CQ/HCQ treatment and a repeat exam at the end of the study to ensure that there was no ocular toxicity.

### Statistical Considerations

The primary endpoint was DLT, and they were defined as dichotomous variables in the study. At each dose level, DLT has been summarized using frequency (%).

Secondary endpoints are a dichotomous variable of treatment response (CR or PR); events of progression free (PF) and overall survival (OS) are both censored at 12 months after treatment. The dichotomous variables of response have been summarized in frequency at each follow-up visit. Kaplan–Meier curves were used to summarize the PFS and OS over time. In addition, as exploratory analyses, logistical and Cox proportional hazard models have been used to assess associations of secondary variables to baseline characteristics such as patient’s demographics, cancer types and stages, and therapy plans.

### Sample Size Justification

Determination of MTD was followed using an algorithm of a maximum of six patients in each cohort. No power analysis was needed as only descriptive statistics are providedfor the primary variables. The analyses of secondary variables were based upon a total of 10 patients in the MDT cohort. Tiered enrollment for each cohort was included according to the standard three to six patients, and it takes up to 28 days to ensure that there are no serious adverse events before moving to the next cohort.

### Data and Safety Monitoring

Review of data and patients’ outcome was discussed at the time of the initiation of the study, before expanding or moving to the following cohort, and at the end of the study. Progress and adverse events were monitored by the University of Cincinnati Cancer Institute Data Safety Monitoring Board after accrual to each dose cohort before approval of accruing to the next cohort.

## Correlative Studies

### Quantification of Autophagosomes

#### From Patients’ Plasma

Patients’ blood samples were collected at the mentioned time points and span down at 1,500*g* for 15 min. The upper phase (plasma) was collected in new tubes and stored at −80°C until use. Exosome’s extraction was done using an exosome extraction reagent (total exosomes precipitation reagent from plasma, Invitrogen by Thermo Fisher Scientific, Ref. 4484451) following the manufacturer’s instructions, then suspended in phosphate-buffered saline (PBS) and stored at −80°C.

### Pharmacokinetics

#### Metabolism of Chloroquine/Hydroxychloroquine

CQ/HCQ was 60% bound to plasma proteins and cleared equally by the kidney and liver. Following administration of C, it was rapidly de-alkylated *via* cytochrome p450 (CYP) into active desethylchloroquine and bisdesethylchloroquine with elimination half-lives of 20–60 days. Both parent drug and metabolite can be detected in urine months after a single dose.

CQ/HCQ has a rapid and almost complete absorption, and peak plasma concentrations reached within 1–2 h following oral administration. CQ/HCQ has a long half-life of 3–5 days. For pharmacokinetic analysis, blood samples (5 ml per time point) were collected on day −7 at baseline pre-dose, then at 1, 2, 4, 6, and 24 h on day 1. Trough levels were collected at days 8 and 15. Blood samples were collected at each subsequent cycle (cycles 2–4) on day 1 at 1, 2, 4, 6, 24, 48, and 72 h. Trough levels were collected on days 8 and 15 for cycles 2–4. Blood was collected into B-D vacutainer tubes containing K3-EDTA mixed and centrifuged at 1,500*g* for 10 min at 4°C. Plasma was transferred into a storage tube and maintained on dry ice until stored in a −20°C freezer. Post-dose trough levels for CQ/HCQ were measured on days 8, 15, and 22 (15).

## Results

### Patients

Twenty-three patients with advanced solid tumors were enrolled between 2014 and 2018. The patient demographic is shown in **Table 2**. Among the 22 eligible treated patients, there were 15 men (68%) and 7 women (32%) with median age of 58 years.

**Table 2:** Patient characteristics.

Cohort/Dose	Tumor Type	Age	Gender	Race	ECOG PS	SAE/AE
1 (CQ) dose level 50 mg	NSCLC-squamous	57	AA	M	1	Neutropenia thrombocytopenia (DLT)
1 dose level 50 mg	NSCLC-adenocarcinoma	41	W	F	2	
1 dose 50 mg	NSCLC	71	W	F	1	
1 dose level 50 mg	GIST	51	W	M	2	Anemia (not DLT)
1 dose level 50 mg	HCC	48	AA	F	1	Diarrhea grade 2
1 dose level 50 mg	Esophageal cancer	55	W	M	1	Anemia (not DLT)
2 (HCQ) dose level 100 mg	HCC	64	AA	F	1	Neutropenia (not DLT)
2 (HCQ) dose level 100 mg	NSCLC-adenocarcinoma	58	W	M	1	
2 dose level 100 mg	HCC	68	AA	F	1	Fatigue (not DLT), nausea, vomiting, thrombocytopenia (grade 2)
3 (HCQ) dose level 150 mg	Urothelial carcinoma	84	Asian	M	0	Fatigue, rash grade I, HTH grade 2
3 (HCQ) dose level 150 mg	Cholangiocarcinoma	68	W	M	1	Neutropenia
3 (HCQ) dose level 150 mg	Refractory SCLC	51	W	M	1	
Expansion (HCQ) dose level 100 mg	NSCLC	61	W	M	2	
Expansion (HCQ) dose level 100 mg	Head and neck cancer	55	W	M	0	
Expansion (HCQ) dose level 100 mg	Metastatic rectal cancer	66	AA	M	1	
Expansion (HCQ) dose level 100 mg	Metastatic colorectal cancer	43	W	M	1	
Expansion (HCQ) Dose Level 100 mg	Other	47	W	F	1	
Expansion (HCQ) dose level 100 mg	Other	65	W	M	0	
Expansion (HCQ) dose level 100 mg	Metastatic adenocarcinoma	57	W	M	0	
Expansion (HCQ) dose level 100 mg	Metastatic adenocarcinoma	61	W	M	0	
Expansion (HCQ) dose level 100 mg	Other	61	W	F	1	
Expansion (HCQ) Dose level 100 mg	Other	57	AA	F	0	
**Variable**		**Number (N)**			**Percentage %**	
**Age in years (median–range)**		**Median**			**Range**	
		**58**			**41–84**	
Gender						
Male		15			68	
Female		7			32	
Race White (W)		15			68	
African American (AA)		6			27	
Asian (A)		1			5	
ECOG PS 0		5			22	
1		14			64	
2		3			14	
Histology						
Non-small cell lung cancer, adenocarcinoma		5			23	
Non-small cell lung cancer, squamous cell carcinoma		4			18	
Other (small cell, urothelial, hepatocellular, and cholangiocarcinoma)		13			59	
Number of prior regimens						
0		3			14	
1		5			22	
2		3			14	
≥3		11			50	

There 15 White (68%), 6 African American (27%), and 1 Asian patient (5%). Regarding ECOG performance status (PS), 5 patients had PS of 0 (22%), 14 had PS of 1 (64%) and 3 had PS of 2 (14%). Tumor histological types were as follows: 5 patients had adenocarcinoma (23%), 4 had squamous cell carcinoma (18%), while 13 had different types (59%) including small cell, urothelial, hepatocellular, and cholangiocarcinoma. The number of regimens received prior to inclusion in this trial was 0 for 3 patients (14%), 1 for 5 patients (22%), 2 for 3 patients (14%), and 3 or more regimens for 11 patients (50%) (**Tables 3**, **4**).

**Table 3:** Clinical outcome.

Outcome	Number of patients (N)	Percentage (%)
**Response rate**		
PR	1	
SD	15	5
PD	6	68
		27
**Disease control**		
**Rate**		
>6 months		48
>12 months		21
>18 months		14

**Table 4:** Table of adverse events.

Event	Grade 2	Grade 3	Grade 4	Grade 5	Total all grades (N)
Fatigue	1	1			2
Rash	1				1
Dehydration		1			1
Leucopenia (Persistent > 7 days)	1 (Baseline)	3			4
Neutropenia (persistent >7days)	1 (Baseline)	3	5		9
Anemia (persistent >7 days)		3	1		4
Thrombocytopenia (persistent >7 days)		2	1		3
Elevated transaminases		2			2
Elevated serum creatinine		1			1
Hyponatremia		1			1
Pain		4 (Unrelated)			4
Weakness		1			1

### Dose escalation

The first cohort constituted of seven patients, as the first patient was excluded and not treated on day 1 and did not meet the eligibility criterion having a baseline platelet count <100,000/µl. Cohort 1 was expanded to include six patients due to treatment-related neutropenia and thrombocytopenia. It was recommended by the Data Safety Monitoring Board to decrease the dose of gemcitabine from 1,250 to 1,000 mg/m^2^. The next three enrolled patients tolerated carboplatin AUC = 5 and gemcitabine 1,000 mg/m^2^ days 1 and 8 in addition to CQ 50 mg with no DLT. HCQ replaced CQ due to an international shortage of CQ at that time. The second cohort included three patients who were treated with HCQ 100 mg daily with no DLT. The first two cohorts of that study thus showed no DLTs at doses of 50 and 100 mg of CQ and HCQ subsequently. The third patient cohort included three patients treated with HCQ 150 mg daily, and two of them experienced DLT due to grade 4 thrombocytopenia and grade 3 neutropenia of more than 7 days duration. The patient with neutropenia did not receive growth factor support. There were no protocol-related deaths.

One DLT occurred in two patients treated with HCQ 150 mg qd group, and the MTD for this combination was determined to be HCQ 100 mg daily. Subsequently, 10 patients were enrolled in the expansion cohort at HCQ 100 mg with carboplatin and gemcitabine.

### Efficacy

**Figure 1:**
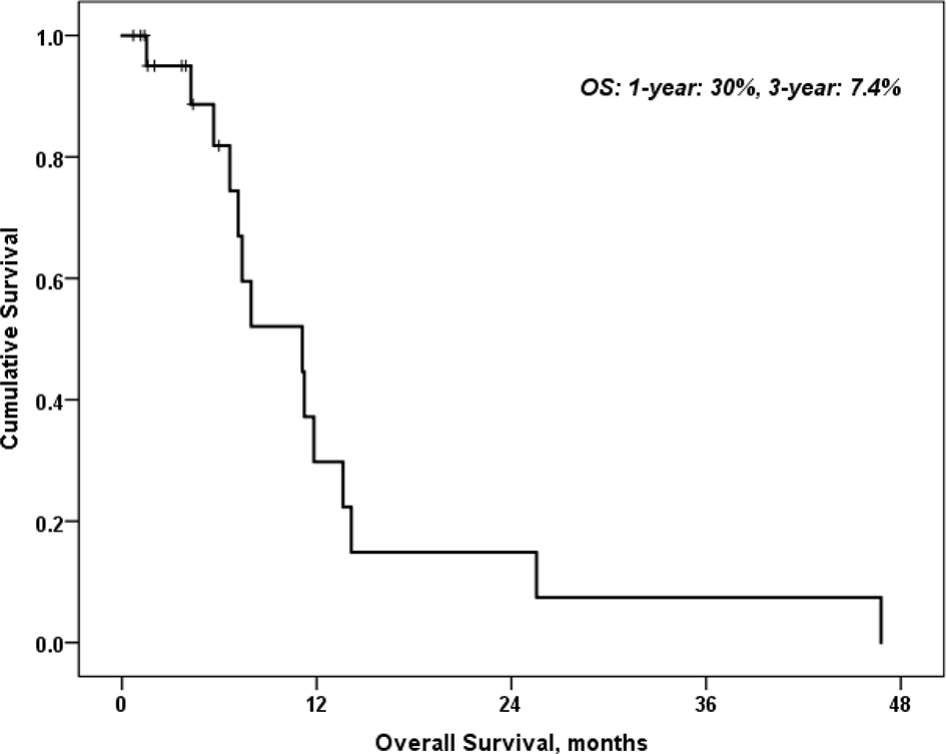
Overall survival of patients with HCQ and carboplatin and gemcitabine.

While assessing the response rate (RR) for various patients included in this study, 1 patient achieved partial response (PR) (5%), 15 patients had stable disease (SD) (68%), while 6 patients had progressive disease (PD) (27%). Nevertheless, the disease control rate (DCR) was 48% for more than 6 months duration, 21% for more than 12 months, and 14% for more than 18 months. In the univariate analysis of predictors of all-cause mortality and predictors of disease progression, neither age, gender, nor number of cycles was statistically significant. Overall, the response rate was 71%. PFS was 48% at 6 months. The DCR was 68% at 6 months, and median overall survival (OS) was 30% at 1 year (**Figure 1**).

### Efficacy of Subsequent Therapies

**Figure 2:**
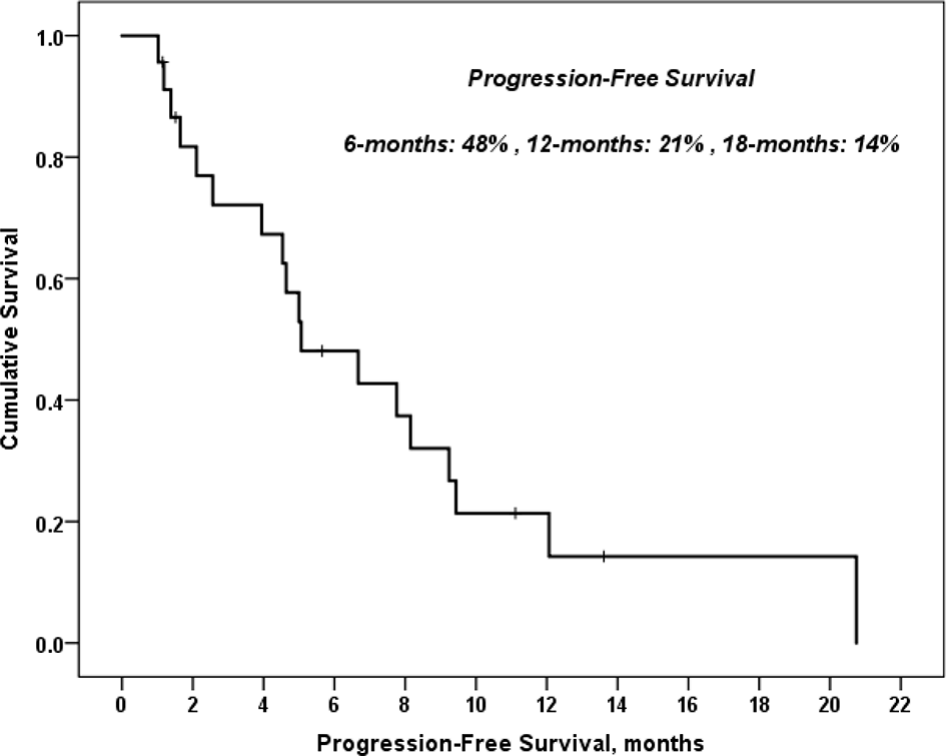
Progression free survival of patients with HCQ and carboplatin and gemcitabine.

Interestingly, we observed that patients receiving subsequent immunotherapy after progressing on this clinical trial had excellent clinical outcomes. One patient with squamous cell carcinoma of the lung (cohort 1) had prolonged stable disease of 11 months on carboplatin and gemcitabine + HCQ. Similarly, prolonged stable disease was noted in a patient with small cell lung cancer in cohort 3 who experienced disease progression on this protocol but then benefited from subsequent nivolumab therapy with a partial remission and improvement of performance status from ECOG 2 to 0. This patient had an ongoing response following 15 cycles of the PDL-1 inhibitor. Another elderly patient in cohort 3 with progressive urothelial cancer tolerated the protocol treatment well with no serious adverse events. This patient achieved disease control with subsequent atezolizumab therapy (**Figure 2**).

## Laboratory Correlates

To assess the effects of treatment on the autophagy pathway, we developed a panel of relevant assays.

### Quantification of Autophagosomes

#### Study Population

This study included 24 patients who were recruited in 4 cohorts, namely, cohort 1 (n=6), cohort 2 (n=3), cohort 3 (n=3), and extension cohort (n=8). All patients were histologically diagnosed with advanced solid. All subjects provided a written informed consent before treatment in accordance with the Declaration of Helsinki, and the study protocol was approved by the Institutional Review Board of the University of Cincinnati Hospital. The subjects enrolled have failed their previous lines of treatment, and the proposed chemotherapy regimen (carboplatin/gemcitabine) was considered a standard of care

### Exosome extraction

#### From Patients Plasma

Patients’ blood samples were collected at the mentioned time points and span down at 1,500*g* for 15 min. The upper phase (plasma) was collected in new tubes and stored at −80°C until use. Exosome’s extraction was done using an exosome extraction reagent (total exosomes precipitation reagent from plasma, Invitrogen by Thermo Fisher Scientific, Ref: 4484451) following the manufacturer’s instructions, then suspended in PBS and stored at −80°C.

#### Western blotting

Detection of LC3b expression in the isolated exosomes was done using Western blotting following standard protocols. LI-COR detection was used to scan the membranes. LC3B protein detection was achieved by anti-LC3B rabbit monoclonal antibody (Cell Signaling Inc., catalogue #2775, USA). CD9 was used as a loading control and was blotted using rabbit monoclonal antibody (#3700) from Cell Signaling Technology Inc. All Western blots were run on 4–15% gradient gels after estimating and unifying sample protein content by bicinchoninic acid (BCA).

LC-3B conversion from LC-3B I to II has been used as an indicator for autophagy, since it measures the dynamicity of the process by reflecting the turnover of autophagosome fusion with lysosomes (16). However, increased expression of both isoforms is used to measure the activity of both autophagy inducers and inhibitors (17, 18).

This methodology is simple and cost effective, which may track stimulus effect on autophagy by interpretation of Western blotting compared to well-known controls.

## Pharmacokinetics

### Metabolism of Chloroquine/Hydroxychloroquine

CQ/HCQ was 60% bound to plasma proteins and cleared equally by the kidney and liver. Following administration of C, it was rapidly de-alkylated *via* cytochrome p450 (CYP) into active desethylchloroquine and bisdesethylchloroquine with elimination half-lives of 20–60 days. Both parent drug and metabolite can be detected in urine months after a single dose.

CQ/HCQ has a rapid and almost complete absorption and peak plasma concentrations reached within 1–2 h following oral administration. CQ/HCQ has a long half-life of 3–5 days. For pharmacokinetic analysis, blood samples (5 ml per time point) were collected on day −7 at baseline pre-dose, then at 1, 2, 4, 6, and 24 h on day 1. Trough levels were collected at days 8 and 15. Blood samples were collected at each subsequent cycle (cycles 2–4) on day 1 at 1, 2, 4, 6, 24, 48, and 72 h. Trough levels were collected on days 8 and 15 for cycles 2–4. Blood was collected into B-D vacutainer tubes containing K3-EDTA mixed and centrifuged at 1,500*g* for 10 min at 4°C. Plasma will be transferred into a storage tube and maintained on dry ice until stored in a −20°C freezer. Post-dose trough levels for CQ/HCQ were measured on days 8, 15, and 22 (15).

Pharmacokinetic (PK) estimates were determined using drug concentrations measured at each time point after HCQ dosing. The maximum plasma concentration (Cmax), time to maximum concentration (Tmax), and area under the concentration–time curve from 0 to 360 h post-dose (AUC0-360h) were determined for the subject following an oral dose. AUC0-360h was calculated using the trapezoidal rule with the linear trapezoidal linear interpolation method. The terminal elimination half-life for each participant was calculated using the last three data points.

For analysis of plasma HCQ, 100 ml of each sample was placed in a low-retention microcentrifuge tube (Thermo Fisher) and spiked with 500 ng of D4-hydroxychloroquine (D4-HCQ; Cayman Chemicals, Ann Arbor, MI). Acetonitrile (300 ml) was added, and the samples were agitated using a vortex mixer for 1 min. The samples were then centrifuged at 10,000*g* for 5 min at 440°C, and 250 ml of the supernatant was transferred to glass high-performance liquid chromatography (HPLC) tubes for mass spectroscopic analysis. Mass spectroscopic acquisition was performed with an ABSciex TripleTOF 5600 (ABSciex, Foster City, CA, USA) equipped with an electrospray interface with a 50-mm i.d. capillary and coupled to an Eksigent μUHPLC (Eksigent, Redwood City, CA, USA). Analyst TF 1.7 software was used to control the instrument and for data processing and acquisition. The optimized MRM parameters were used to monitor HCQ and D4-HCQ. HCQ, the parent ion, was 337.2, and the selected MRM MS/MS ion was 248.15. For D4-HCQ, the parent ion was 341.2, and the selected MRM MS/MS ion was 252.12. Separation was performed on a reversed-phase ACE C18 50 mm × 0.5 mm column, which was maintained at 450°C. Samples were injected by loop overfilling into a 2-ml loop. For the 2.5-min LC gradient, the mobile phase consisted of solvent A (0.1% v/v formic acid in water) and solvent B (0.1% v/v formic acid in acetonitrile) at a flowrate of 40 ml/min. Gradient started from 95:5 A:B. Data integration and quantification were performed with MultiQuant software (ABSciex) using the area under the curve (**Tables 5**, **6**).

**Table 5:** Pharmacokinetics of patients 9–13 in cycle 1.

	Cycle 1							
Time	9	10	11	12	13	Mean	SD	SEM
**D-7**	0.0	0.0	102.1	0.0	0.0	20.4	45.7	20.5
**D1 1h**	206.0	46.2	82.6	161.2	123.4	123.9	63.0	28.2
**D1 2h**	263.5	75.4		172.1	150.8	165.5	77.4	38.7
**D1 4h**	320.5	70.7	224.6	183.3	174.5	194.7	90.3	40.5
**D1 6h**	229.0	57.1	235.7	192.4	116.4	166.1	77.2	34.6
**D1 24h**	160.5	22.1		78.6	126.7	97.0	60.2	30.1
**D1 48h**			58.2			58.2		0.0
**D1 72h**								
**D8**	101.0	38.3	119.6	175.2	32.1	93.2	59.7	26.7
**D15**	57.0	7.5		93.4	0.0	39.5	43.9	22.0

SD, Standard deviation, SED, Standard error of mean.

**Table 6:** Pharmacokinetics of patients 9–13 in cycle 2.

	Cycle 2							
Time	9	10	11	12	13	Mean	SD	SEM
**D-7**								
**D1 1h**		60.3	29.7	220.6		103.6	102.5	59.3
**D1 2h**		109.6	37.8	250.7		132.7	108.3	62.6
**D1 4h**		88.9	25.7			57.3	44.7	31.7
**D1 6h**		105.3	22.7	239.4		122.4	109.4	63.2
**D1 24h**		97.2	20.5	50.2		56.0	38.7	22.4
**D1 48h**			71.9	224.8		148.3	108.1	76.7
**D1 72h**			49.2	380.7		214.9	234.4	166.3
**D8**		64.1	58.9	122.6		81.9	35.4	20.4
**D15**			44.9	53.7		49.3	6.2	4.4

SD, Standard deviation, SED, Standard error of mean.

## Discussion

Our study determined an MTD for HCQ that was very close to the dose determined in a study using CQ in addition to the standard therapy for patients with glioblastoma multiforme (5).

One limitation of our study is that we used HCQ instead of CQ for the continuation of the study due to the acute shortage of CQ in the US and the inability of our institution to obtain CQ.

Our study used CQ or HCQ combined with carboplatin and gemcitabine (CG) in a heavily pretreated patient population with various advanced solid tumors. As a result, the MTD appeared to be much lower than the MTD dose of CQ or HCQ in reported other studies (19). The highest-dose cohort in the current study included patients who were heavily pretreated and experienced >7 days of either neutropenia or thrombocytopenia. No on-study deaths occurred. Other trials that incorporated either CQ or HCQ were able to deliver higher doses of these agents given that these studies included agents that are not usually myelosuppressive (19) or in chemotherapy-naive subjects (20).

Subsequent responses to immunotherapeutic agents such as PD-1 or PD-L1 inhibitors may be considered in the future. Autophagymodifying agents combined with evolving immunotherapy as a potential new treatment option may offer an interesting area for additional studies, both in the laboratory and clinic. Autophagy is involved in the processing of tumor antigens and their presentation to the immune system and thus may be considered as a line of defense against cancer.

Autophagic pathways induced by hypoxia in the tumor microenvironment can impair antitumor immune responses mediated by cytotoxic T-lymphocytes (CTL) and natural killer (NK) cells and has also been shown to enhance the immunosuppressive properties of myeloid-derived suppressor cells (MDSCs) (20). In response to hypoxia, the hypoxia-inducible family of transcription factors (HIFs) does not become ubiquitinated, thus evading degradation by the ubiquitin–proteasome system. As a result, they accumulate in the cytoplasm and are transported to the cell nucleus leading to the activation of about 300 genes involved in many biological processes, including angiogenesis, enhanced cell survival, metastasis, induction of a stem cell-like phenotype, and immune escape (20). Targeting HIF-2α decreases PD-L1 expression, whereas HIF-2α overexpression increases both PD-L1 mRNA and protein expression in renal cancer cells (21).

In his study, Wolpin et al. used HCQ as monotherapy for previously treated metastatic pancreatic cancer, and he achieved much lower median PFS and OS (46.5 and 69.0 days, respectively) while using higher doses or HCQ (400 and 600 mg twice daily dose) (22). In addition, Malhotra et al. used chemotherapy [carboplatin, paclitaxel (and bevacizumab if meeting criteria)] in addition to HCQ (twice daily dose of 200–600 mg) in newly diagnosed non-small cell lung cancer (NSCLC) patients achieving a PFS of 3.7 months, thus demonstrating an improved response with addition of HCQ even with lower doses to the CG chemotherapy regimen (23).

Barbeau et al. (24) concluded that for increased survival, early or advanced stages are dependent on autophagy. The high metabolic demand and increased resistance to chemotherapeutic agents are dependent on autophagy *via* genetic mutations, such as *EGFR*, *EGFRvIII*, and *BRAFv600E*. Compter et al. (25) have described the role of autophagy in glioblastoma cells expressing *EGFRvIII*. The maximum tolerated dose of CQ was 200 mg. The median overall survival time was 16 months. The median survival of patients with *EGFRvIII*− was 11.5 months and that of patients with *EGFRvIII*+ was 20 months. In their study, a total of 44 adverse events were related to CQ with QT prolongation and blurring of vision along with nausea and vomiting. In our study, the overall median survival was 11 months, and the MTD was 100 mg, with neutropenia and thrombocytopenia as the limiting factors. Levy et al. suggested a role for the molecular mechanisms by which autophagy affects the tumor microenvironment (26).

## Conclusion

The results from this study demonstrate that HCQ opens a new era for heavily treated HCQ-naive patients to receive HCQ in addition to chemotherapy, thus improving both PFS and overall survival. These are still ambitious hypotheses that need further research, and that is why we need to expand our trial to phase II.

The switch from CQ to HCQ had to occur due to the acute shortage of CQ supply during the time of the phase I clinical trial, which might be a limitation to our study.

## Data Availability Statement

The original contributions presented in the study are included in the article/supplementary material. Further inquiries can be directed to the corresponding author.

## Ethics Statement

The studies involving human participants were reviewed and approved by University of Cincinnati. The patients/participants provided their written informed consent to participate in this study.

## Author Contributions

All authors listed have made a substantial, direct, and intellectual contribution to the work and approved it for publication.

## Funding

This trial was funded by the Division of Hematology/Oncology, Department of Internal Medicine, University of Cincinnati. Institutional Support from The University of Cincinnati-Division of Hematology Oncology EXP-1301 clinical trial.

## Conflict of Interest

Author AK was employed by GlaxoSmithKline.

The remaining authors declare that the research was conducted in the absence of any commercial or financial relationships that could be construed as a potential conflict of interest.

## Publisher’s Note

All claims expressed in this article are solely those of the authors and do not necessarily represent those of their affiliated organizations, or those of the publisher, the editors and the reviewers. Any product that may be evaluated in this article, or claim that may be made by its manufacturer, is not guaranteed or endorsed by the publisher.
